# Regenerative Drug Discovery Using Ear Pinna Punch Wound Model in Mice

**DOI:** 10.3390/ph15050610

**Published:** 2022-05-16

**Authors:** Paweł Sosnowski, Piotr Sass, Paulina Słonimska, Rafał Płatek, Jolanta Kamińska, Jakub Baczyński Keller, Piotr Mucha, Grażyna Peszyńska-Sularz, Artur Czupryn, Michał Pikuła, Arkadiusz Piotrowski, Łukasz Janus, Sylwia Rodziewicz-Motowidło, Piotr Skowron, Paweł Sachadyn

**Affiliations:** 1 Laboratory for Regenerative Biotechnology, Gdańsk University of Technology, 80-233 Gdańsk, Poland; paw.sosno@gmail.com (P.S.); piotrsass@gmail.com (P.S.); slonimska.p@gmail.com (P.S.); rplatek1982@gmail.com (R.P.); jolantakaminska6@gmail.com (J.K.); kellerbaczynski@gmail.com (J.B.K.); 2 Department of Molecular Biochemistry, Faculty of Chemistry, University of Gdańsk, 80-308 Gdańsk, Poland; piotr.mucha@ug.edu.pl; 3 Tri-City University Animal House—Research Service Centre, Medical University of Gdańsk, 80-211 Gdańsk, Poland; gsularz@gumed.edu.pl; 4 Laboratory of Neurobiology, Nencki Institute of Experimental Biology PAS, 02-093 Warsaw, Poland; artur@nncki.gov.pl; 5 Laboratory of Tissue Engineering and Regenerative Medicine, Department of Embryology, Medical University of Gdańsk, 80-211 Gdańsk, Poland; pikula@gumed.edu.pl; 6 Department of Biology and Pharmaceutical Botany, Faculty of Pharmacy, Medical University of Gdańsk, 80-416 Gdańsk, Poland; arpiotr@gumed.edu.pl; 7 MedVentures Company, 60-141 Poznań, Poland; j.medventures@gmail.com; 8 Department of Biomedicinal Chemistry, Faculty of Chemistry, University of Gdańsk, 80-308 Gdańsk, Poland; s.rodziewicz-motowidlo@ug.edu.pl; 9 Department of Molecular Biotechnology, Faculty of Chemistry, University of Gdańsk, 80-308 Gdańsk, Poland; piotr.skowron@ug.edu.pl

**Keywords:** regeneration, regenerative medicine, pharmacoregeneration, regenerative drugs, epigenetic drugs, ear pinna punch wound model

## Abstract

The ear pinna is a complex tissue consisting of the dermis, cartilage, muscles, vessels, and nerves. Ear pinna healing is a model of regeneration in mammals. In some mammals, including rabbits, punch wounds in the ear pinna close spontaneously; in common-use laboratory mice, they remain for life. Agents inducing ear pinna healing are potential regenerative drugs. We tested the effects of selected bioactive agents on 2 mm ear pinna wound closure in BALB/c mice. Our previous research demonstrated that a DNA methyltransferase inhibitor, zebularine, remarkably induced ear pinna regeneration. Although experiments with two other demethylating agents, RG108 and hydralazine, were unsuccessful, a histone deacetylase inhibitor, valproic acid, was another epigenetic agent found to increase ear hole closure. In addition, we identified a pro-regenerative activity of 4-ketoretinoic acid, a retinoic acid metabolite. Attempts to counteract the regenerative effects of the demethylating agent zebularine, with folates as methyl donors, failed. Surprisingly, a high dose of methionine, another methyl donor, promoted ear hole closure. Moreover, we showed that the regenerated areas of ear pinna were supplied with nerve fibre networks and blood vessels. The ear punch model proved helpful in testing the pro-regenerative activities of small-molecule compounds and observations of peripheral nerve regeneration.

## 1. Introduction

Small-molecule regenerative drugs may be a great challenge and an excellent chance for regenerative medicine. The search for new drugs that stimulate regeneration is difficult due to the complexity of regenerative processes. Though infrequently reported, the ear pinna model seems a promising tool for regenerative drug discovery.

**Figure 1:**
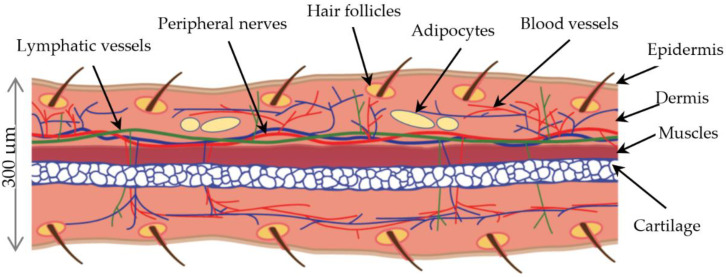
Diagram of mouse ear pinna anatomy.

The auricle, also known as the ear pinna, constitutes the external ear together with the ear canal. It is typical of mammals and absent in other vertebrates. The ear pinna collects, amplifies, and directs sounds to the auditory canal; it performs spectral transformations to incoming sounds and enhances sound localisation [1]. In some animals, the functions of ear pinna are heat radiation and mood signalling [2]. The ear pinna is approximately 300 µm thick in adult mice [3]. The appendage has a complex tissue architecture that involves cartilage, skin, vessels, muscles, and nerves. The structure is supported by an internal sheet of elastic cartilage consisting of two layers of chondrocytes approximately 60 µm thick between two thin layers of skin with few hair follicles. The 25–40 µm-thick epithelium is formed by 2–3 layers of keratinocytes, which are covered by a 10 µm-thick stratum corneum. The 25–60 µm-thick dermis comprises a dense extracellular matrix and scarce elongated fibroblasts [4]. The ear pinna is supplied by a complex system of lymphatic [5] and blood vessels and a rich network of peripheral nerves [6]. The striated muscle fibres are situated between the layers of cartilage and the dorsal dermis [5,7]. Figure 1 presents the diagrammatic anatomy of the mouse ear pinna.

Though auricle injuries do not appear as a challenge to regenerative medicine, the observation, first reported by Markelova in 1953, that punches 1 cm in diameter made in the ear pinna in rabbits close entirely within 8 weeks attracted attention as a model to study regeneration in mammals [8]. As ear punches in rabbits heal with scarring, the model may be used for studying hypertrophic scars [9]. In laboratory mice, where ear punches are made to mark animals, the ear holes remain for life. The discovery published in 1998 that an inbred strain, the MRL mouse, healed 2 mm diameter ear punch holes entirely within 4–5 weeks without scarring [10] incited research on the mechanisms underpinning the process. The remarkable regeneration capacity in the MRL mouse was found to have a multigenic basis [11] and involved the restoration of the dermis, cartilage, muscles, vessels [10], and peripheral nerves [12]. Thus, the model allows the investigation of regenerative responses in complex tissues. The ear pinna model offers a significant advantage—the absence of skin contraction. The cartilage sheet between the dermis layers prevents rapid wound shrinking observed in loose dorsal skin. Therefore, some researchers noticed the skin excision made in the ear pinna as an alternative to the dorsal wound model to study epithelialisation [13]. It is worth pointing out an essential difference between through-and-through ear pinna wounds and full-thickness excisional dorsal skin wounds. In the dorsum, the whole excision surface is considered an open wound, whereas the wounds in the ear pinna can be regarded as the edges around the punch holes. The formation of undifferentiated tissues resembling blastema in healing ear pinnae suggested parallels with epimorphic regeneration [14], the type of regeneration observed during the regrowth of limbs in amphibians, where a mass of undifferentiated cells covers the stump and gives the origin to a new limb [15].

The role of angiogenesis in tissue repair is well established and apparent; vessels are necessary to provide oxygen and nutrients. As shown by denervation experiments both in amphibian limb regeneration and cutaneous regeneration in mammals, regeneration is nerve dependent [16]. The mouse ear pinna offers excellent possibilities to visualise blood vessels and peripheral nerves [6]. These aspects of ear pinna regeneration seem understudied, but ear pinna healing can also be considered a promising model for preliminary testing of neurotrophic and angiogenic agents.

Ear pinna healing is convenient to track and quantitate as the percentage of wound closure. In addition, the injury is not associated with severe stress and pain to animals. Furthermore, the capacity for ear pinna wound closure might be improved through genetic modifications, including both monogenic mutations as in the *Foxn1* [17] or *p21* [18] deficient mice or transgene delivery as in the mouse overexpressing the *Angptl6* gene encoding the AGF protein in epidermal keratinocytes [19]. Since genetic modifications can enhance the regenerative response in the ear pinna, pharmacological treatment should make it possible to induce similar effects. Improved but incomplete ear hole closure has been reported following subcutaneous injections of a hypoxia activator [20] and topical application of a Wnt-signalling inhibitor [21].

The ear pinna model’s essential advantage is that punch wound closure can manifest regenerative potential in other tissues. In the MRL mouse, enhanced regenerative responses have been reported in the heart [22], the spinal cord [23], tendons ([24], the cornea [25], the retina [26], skeletal muscles [27], and digit tips [28]. Similarly, the Foxn1-deficient nude mice show both improved ear pinna and dorsal skin wound healing [17]. *Acomys*, known as the African spiny mouse, closes even 4 mm ear pinna holes. In *Acomys*, the regenerative capacity is not restricted to the ear pinna; the animal displays an unusual ability to heal large excisional wounds in the back without scarring [29].

Our previous research determined associations between gene methylation profiles and regenerative capacity [30,31,32,33]. Next, we found that zebularine, a DNA methyltransferase inhibitor, promoted auricle regeneration, and its combination with all-trans-retinoic acid accelerated the process, leading to complete ear pinnae hole closure. Our data indicate that zebularine-mediated demethylation activates epigenetically silenced genes, and retinoic acid supports their transcriptional induction. What is essential is that the regenerated ear pinna showed the restoration of proper tissue architecture [34]. The present study demonstrates further experimental data on zebularine effects on ear pinna punch healing in mice and tests with several other bioactive compounds, including epigenetic inhibitors, methyl group donors, and immunomodulators. In addition, we analyse the variation observed in the ear pinna hole closure and the impact of age and reveal the development of the nerve fibre networks in regenerating ear pinnae.

## 2. Results and Discussion

### 2.1. Delayed Zebularine Delivery

**Figure 2:**
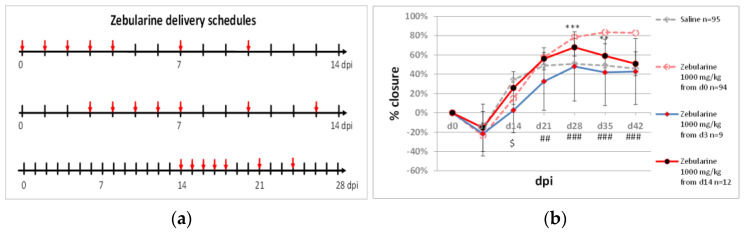
The effect of 3- and 14-day delays in zebularine administration on ear pinna hole closure. (**a**) Zebularine delivery schedules; arrows indicate the days of injection. (**b**) The time plots of ear pinna hole closure for zebularine treatments started on d0, d3, and d14 post-injury. Significant differences between zebularine administration started on d3, and d0 below 0.01 and 0.001 are indicated with double and triple hashtags (##, ###), respectively. A significant difference between zebularine administration started on d3 and saline receiving controls below 0.05 is marked with a dollar sign ($). Significant differences between zebularine administration started on d14 and d0 are pointed out with a double and triple asterisk (**, ***), respectively. n represents the number of wounds (ears); dpi—days post injury.

Our previous research reported approximately 83% closure of ear pinna punch wounds following intraperitoneal zebularine injections (1000 mg/kg) [34]. The treatment consisted of seven injections, where the first one was administered immediately after the injury and the last one on day 10 post-injury. Improved healing was recorded from day 21, and ear pinna closure was inhibited on days 7 and 14 post-injury. This observation provoked the question of whether shifting zebularine treatment by several days from the day of injury may abolish this initial negative effect. We performed two experiments with the first zebularine dose applied on day 3 or 14 after the injury (Figure 2a). A 72 h delay in commencing the zebularine treatment resulted in a complete loss of the healing effect. In addition, a significant reduction in wound closure compared to saline controls was observed on day 14 post-injury. Similarly, the healing effect was lost when zebularine treatment commenced on day 14 after the injury, though ear hole closure was transiently improved compared to the saline-treated controls on days 28 and 35 (Figure 2b).

The results show that the time immediately following the injury is critical for the pro-regenerative activity of zebularine. The first 72 h post-injury were within the inflammatory phase of healing, suggesting that zebularine may modulate immune responses to wounding, which corresponds with a report on the immunosuppressive activity of zebularine [35]. Interestingly, zebularine delivery started on day 14 post-injury improved ear hole closure compared to the saline controls as recorded for days 28 and 35. This observation indicates that zebularine delivery can still promote the growth of ear pinna tissues even several days after the inflammatory phase is terminated and wound edges sealed.

### 2.2. Modifying Zebularine Effects with Small-Molecule Bio-Active Compounds

Combining zebularine delivery with all-trans-retinoic acid results in accelerated and almost complete ear pinna hole closure compared to zebularine applied alone [34]. Below, we present experiments with several other compounds applied to enhance zebularine’s healing effects.

#### 2.2.1. Immunomodulators

**Figure 3:**
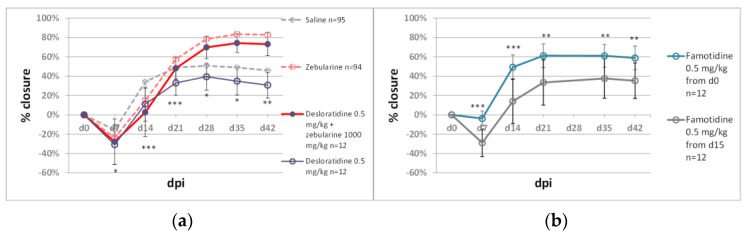
The effect of H1 and H2 blockers, (**a**) desloratidine and (**b**) famotidine, on zebularine-mediated ear pinna hole closure. Single, double, and triple asterisks (*, **, ***) denote statistical significance below 0.05, 0.01, and 0.001, respectively. n represents the number of wounds (ears); dpi—days post injury.

**Figure 4:**
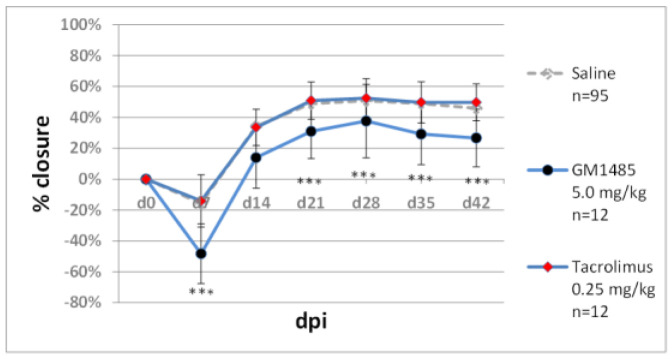
The effect of immunophilin ligands GM1485 and tacrolimus on ear pinna hole closure. Triple asterisks (***) denote statistical significance below 0.001. n represents the number of wounds (ears); dpi—days post injury.

Wound healing and regeneration depend on immune responses. Inflammation is an important phase of healing beginning immediately after the injury, whereas prolonged injury inhibits tissue repair [36,37]. Therefore, immunomodulators are likely to impact the regenerative process. For preliminary tests in the ear pinna model, we selected histamine blockers, desloratidine and famotidine, and immunophilin ligands, tacrolimus and G1485. Desloratidine (0.5 mg/kg), an H_1_-blocker, showed a negative effect on wound closure compared to the saline controls, but it did not significantly reduce zebularine action (Figure 3a). Famotidine, an H_2_-receptor antagonist, showed a moderately positive but statistically significant impact on ear hole closure, though it did not augment the action of zebularine (Figure 3a). Starting famotidine administration on day 14 post-injury abolished its positive effects (Figure 3b). Tacrolimus inhibits calcineurin, which is, in turn, involved in the production of interleukin 2, one of the critical molecules in immune signalling [38]. Tacrolimus treatment (0.25 mg/kg) did not affect ear pinna healing. GM1485 is a non-immunosuppressive immunophilin ligand not interacting with calcineurin and has already been reported to promote neural regeneration following i.p. injections at 5 mg/kg [39]. GM1485, used at the same dose in our model, significantly decreased ear pinna wound closure (Figure 4).

The tested immunomodulators administered at the indicated doses did not add to the zebularine effect on ear pinna healing. However, famotidine showed a moderate improvement in ear hole closure. Though the preliminary experiments with immunomodulators were not encouraging, combining epigenetic inhibitors with immunomodulators to potentiate regenerative responses seems to deserve further focus.

#### 2.2.2. Methyl Donors

Zebularine is a demethylating agent [40], and its pro-regenerative action has been associated with global and gene-specific decreases in DNA methylation in the wound area [34]. Therefore, methyl donors may be expected to counteract the demethylating and thus the pro-regenerative activity of zebularine. On the other hand, folate intervention has been found to induce regeneration of afferent spinal neurons in rats following intraperitoneal administration at 0.08 mg/kg [41]. Methyl donors may also be helpful to investigate the mechanisms underpinning the regenerative effects of zebularine.

**Figure 5:**
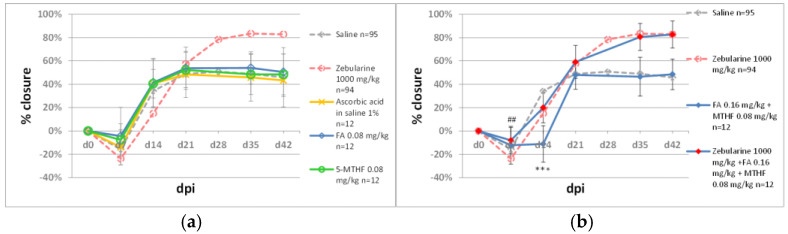
The effect of folates on ear pinna hole closure. (**a**) Impact of folic acid (FA) and 5-methyltetrahydrofolate (5-MTHF); (**b**) impact of combinational administration of FA and MTHF on zebularine-induced ear pinna healing. A triple asterisk (***) denotes statistical significance between folates and saline below 0.001. A double hashtag (##) indicates statistical significance between zebularine+folates and folates below 0.01. n represents the number of wounds (ears); dpi—days post injury.

**Figure 6:**
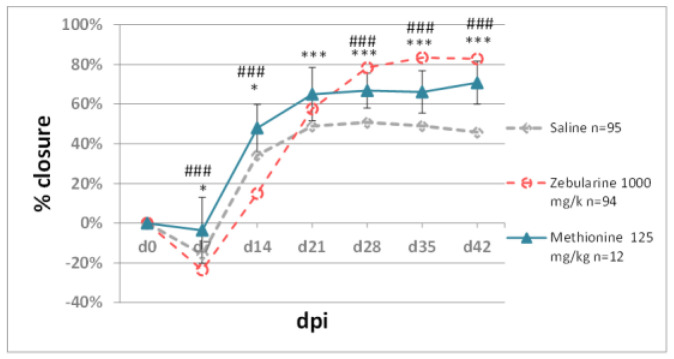
The effect of high-dose methionine on ear pinna hole closure. Single and triple asterisks (*, ***) denote statistical significance between methionine and saline below 0.05 and 0.001, respectively; triple hashtags (###) indicate statistical significance between methionine and zebularine below 0.001. n represents the number of wounds (ears); dpi—days post injury.

Our experiments used dietary donors of the methyl group folic acid; its bioavailable form, 5-methyltetrahydrofolate; and methionine. In the ear pinna model, neither folic acid (0.08 mg/kg) nor 5-methyltetrahydrofolate (0.08 mg/kg) affected wound closure (Figure 5a). Joint treatment with folic acid (0.016 mg/kg) and methyltetrahydrofolate (0.08 mg/kg) showed no effect on ear pinna closure, except on day 14 post-injury when it was transiently inhibited compared to the saline-treated controls. The folates did not impact zebularine-mediated ear pinna healing (Figure 5b). Methionine (125 mg/kg) significantly improved ear pinna hole closure; however, the effect did not equal that of zebularine (Figure 6).

Iskandar et al. demonstrated that intraperitoneal folate injections at 0.08 mg/kg promoted nerve repair in rats, whereas higher doses (0.16–0.80 mg/kg) decreased DNA methylation levels in the injured spinal cord [41]. In our experiments, folates showed no effect on ear pinna healing at 0.08 mg/kg. Folates at 0.24 mg/kg transiently delayed ear pinna hole closure. An attempt to counteract the healing effect of the demethylating agent zebularine, with folates as the methyl group donors, proved unsuccessful. Of note, the approximate dietary folate intake in mice is 10 µg daily [42], corresponding to 0.4–0.5 mg/kg.

Methionine is another methyl group donor we examined in the ear pinna wound model. Methionine has been already used successfully as a methyl donor to reverse epigenetic changes [43]. A high methionine dose (125 mg/kg) applied in our experiment significantly promoted ear pinna hole closure. Therefore, we did not try methionine to thwart the demethylating and pro-regenerative action of zebularine. The daily intake of dietary methionine could be estimated as approximately 0.8 mg/kg, assuming 3.2 µg per 1 g of standard maintenance mouse diet (C1320, Altromin) and 5 g feed consumption per mouse weighing 20 g. A dose of 125 mg/kg exceeds the daily dietary intake enormously (over 150-fold), thus suggesting that the dietary methionine intervention to promote regeneration may not be tenable. Methionine in high doses is likely to induce DNA demethylation by inhibiting homocysteine remethylation and increasing S-adenosylhomocysteine (SAH) levels. Increased SAH levels lower the activity of DNA methyltransferases, thus leading to DNA demethylation [44]. Methionine-mediated ear pinna healing presents an exciting option for further investigations on this essential amino acid as a DNA demethylating and pro-regenerative agent.

### 2.3. Testing Retinoids and Vitamin D_3_ in the Ear Punch Wound Model

**Figure 7:**
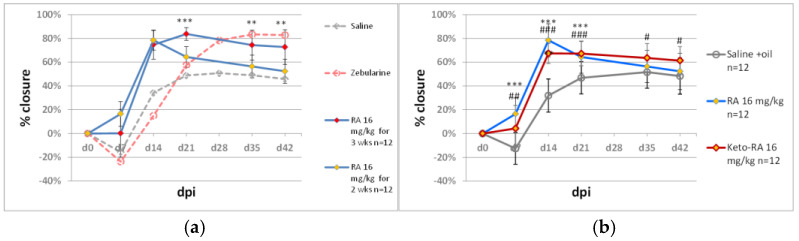
The effect of retinoids on ear pinna hole closure. (**a**) Comparison of 2- and 3-week treatment with retinoic acid (RA); (**b**) comparison of retinoic acid (RA) and 4-ketoretinoic acid (keto-RA) effects. Double and triple asterisks (**, ***) denote statistical significance between RA and controls below 0.01 and 0.001, respectively. Single, double, and triple hashtags (#, ##, ###) indicate statistical significance between keto-RA and control below 0.05, 0.01, and 0.001, respectively. n represents the number of wounds (ears); dpi—days post injury.

Retinoids are known for their role in regenerative processes [45] and their importance for skin functions [46]. Vitamin D_3_ has been reported to stimulate neuronal, vascular, and muscle regeneration [47,48,49]. As mentioned above, we demonstrated that retinoic acid (all-trans-retinoic acid) dramatically potentiated the effect of zebularine on ear pinna regeneration. When applied alone, retinoic acid moderately improved ear pinna hole closure. [34]. In the present study, we examined the pro-regenerative activity of 4-ketoretinoic acid (all-trans 4-keto retinoic acid), a metabolite of retinoic acid. 4-ketoretinoic acid (16 mg/kg) showed a similar and, at days 35 and 42 post-injury, slightly better effect on ear pinna hole closure than retinoic acid (Figure 7a). Unlike zebularine, retinoic acid treatment (16 mg/kg, for two weeks, five doses per week) resulted in a maximal ear hole closure at day 14 post-injury, followed by increasing ear holes at the subsequent time points. The extension of retinoic acid treatment to the third week with three injections a week greatly increased the closure effect, with a result similar to that obtained with zebularine (1000 mg/kg) (Figure 7b).

**Figure 8:**
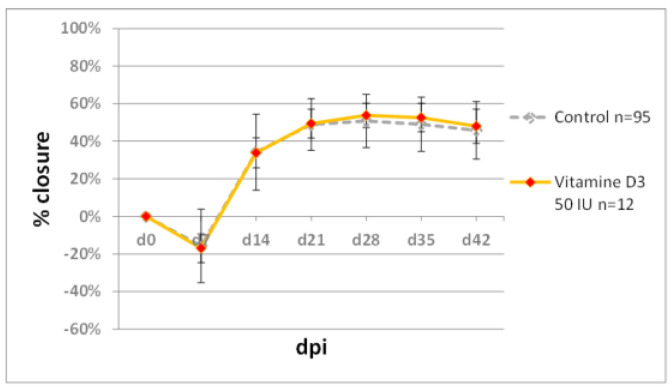
The effect of orally delivered vitamin D_3_ on ear pinna hole closure. Vitamin D_3_ was delivered per os in 10 doses of 50 IU, each administered within 2 weeks according to the schedule described in Materials and Methods, Section 3.2. n represents the number of wounds (ears); dpi—days post injury.

There was no improvement in ear pinna hole closure following two-week treatment with vitamin D_3_ per os (Figure 8). The applied dose of 50 IU per day exceeded 16-fold the daily dietary intake of 3 IU. (The dose of 3 IU per mouse was calculated assuming 0.6 IU per 1 g of standard maintenance feed (C1320, Altromin) and 5 g feed consumption daily).

A significant increase in ear pinna hole closure was obtained by extending retinoic acid administration from 2 to 3 weeks. This result indicates that retinoic acid actively stimulates ear pinna healing within the third week post-injury, and this time interval is critical for the repair effect. The observation that 4-ketoretinoic acid promoted ear pinna hole closure similar to retinoic acid is in line with the findings that 4-ketoretinoic acid is not only the product of the retinoic acid inactivation pathway but an active signalling molecule [50]. There are no literature data to compare the toxicity of retinoic and 4-ketoretinoic acid directly, but the latter, as the product of retinoic acid metabolism, is likely to be better tolerated by the organism. The applied dose of retinoic acid (16 mg/kg) was markedly lower than the LD50 value of 790 mg/kg i.p. reported in mice [51], but the risk of toxicity and teratogenicity should be considered in treatments involving retinoic acid. Retinoic acid may cause liver damage, especially when combined with other drugs [52]. The topical application of retinoic acid displays a much better safety profile, although partial systemic penetration occurs [53]. The effect of retinoid administration on ear pinna regeneration corresponds with the report on transcriptional activation of retinoic acid metabolism genes in the MRL mouse, the strain known for its innate ability to regenerate ear pinna wounds [54]. 

### 2.4. Diet and Ear Pinna Hole Closure

**Figure 9:**
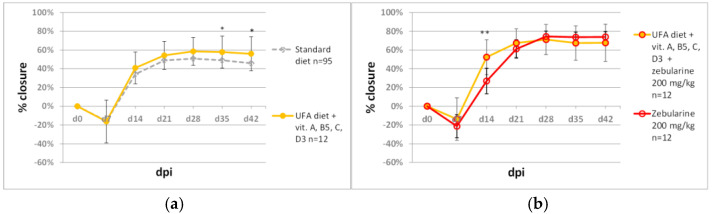
The effect of an unsaturated fatty acid-enriched diet (UFA diet) fortified with vitamins A, B_5_, C, and D_3_ on zebularine-mediated ear pinna hole closure. (**a**) UFA vitamin-fortified vs standard diet; (**b**) UFA vitamin-fortified diet impact on zebularine-induced ear pinna hole closure. Single and double asterisks (*, **) indicate statistical significance below 0.05 and 0.01, respectively. n represents the number of wounds (ears); dpi—days post injury.

Diet impacts the organism’s condition, and dietary factors are likely to modulate the effect of regenerative therapies. For our tests, we selected a fat-rich diet enriched in unsaturated fatty acids [55,56] fortified with vitamins A, B_5_, C, and D_3_ (Table 1). The diet alone did not strongly affect ear hole closure, although slight statistically significant increases were observed on days 35 and 42 (Figure 9a). The diet did not enhance the final result of ear hole closure induced by zebularine treatment (200 mg/kg) but markedly neutralised the inhibitory effects of zebularine in the beginning phase of ear hole closure, as recorded on day 14 post-injury (Figure 9b).

The impact of the tested diet on ear pinna hole closure was not remarkable, although statistically significant improvements were recorded. The observations show that dietary supplementation helps investigate the nutritional effects on healing. Noteworthy, the dietary intervention was limited to a short period of 14 days post-injury in the presented experiments, and it would be warranted to investigate whether long-term dietary supplementation can intensify the regenerative responses. It should be stressed that the experiment on the diet effect on regeneration presented here is preliminary. Optimising the pro-regenerative diet and explaining the mechanisms of its actions deserves a focused study.

### 2.5. Testing Non-Nucleoside Epigenetic Inhibitors in the Ear Punch Wound Model

**Figure 10:**
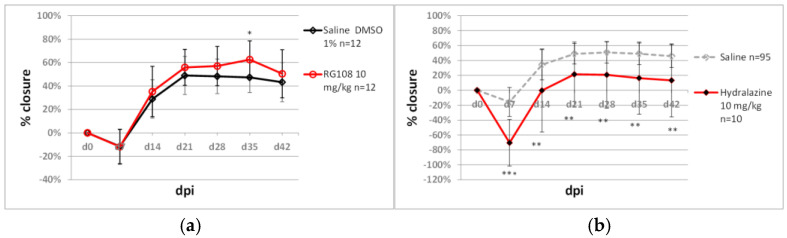
The effect of non-nucleoside DNA methyltransferase inhibitors on ear pinna hole closure. (**a**) RG108 delivered in saline with 1% DMSO (saline with 1% DMSO was used as the control); (**b**) hydralazine. Single, double and triple asterisks (*, **, ***) denote statistical significance below 0.05, 0.01 and 0.001, respectively. n represents the number of wounds (ears); dpi—days post injury.

**Figure 11:**
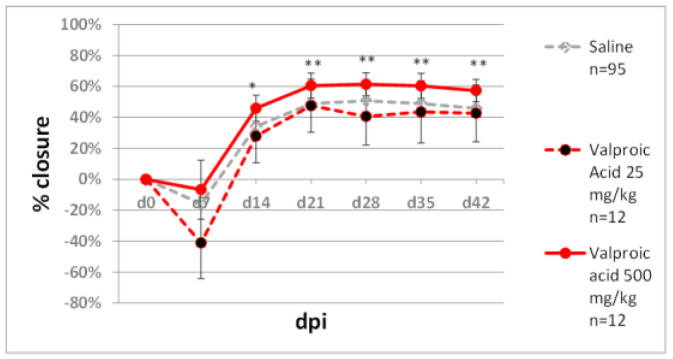
The effect of valproic aid, a histone deacetylase inhibitor, on ear pinna hole closure. Single and double asterisks (*, **) indicate statistical significance below 0.05 and 0.01, respectively. n represents the number of wounds (ears); dpi—days post injury.

Zebularine, a nucleoside shown to promote tissue regeneration [34], displays minimal toxicity in cellular [57] and animal models [58]. However, zebularine’s demethylating activity requires incorporation into DNA, which may cause mutations [59,60]. Therefore, we decided to examine whether ear pinna regeneration can be stimulated using non-nucleoside epigenetic inhibitors. The experiments involved RG108 and hydralazine. The first is a selective DNA methyltransferase inhibitor [61], the latter an antihypertensive drug lowering DNA methyltransferase levels by inhibiting mitogen-activated protein kinase [62]. RG108 (10 mg/kg) did not induce marked ear hole closure, but a slight improvement compared to the controls was recorded on day 35 post-injury (Figure 10a). Hydralazine (10 mg/kg) deteriorated ear pinna hole closure (Figure 10b). Another epigenetic inhibitor tested was valproic acid, an antiepileptic drug acting as a histone deacetylase inhibitor. A moderate dose of 25 mg/kg of valproic acid demonstrated no effect on ear pinna hole closure. Applying a high dose of 500 mg/kg resulted in a modest but statistically significant ear hole closure improvement compared to the controls (Figure 11).

Hydralazine has been shown to prevent fibrosis in a murine model of acute kidney injury-to-chronic kidney disease progression administered intraperitoneally at 5 mg/kg below the blood pressure-lowering dose of 50 mg/kg [63]. RG108 has been demonstrated to induce DNA demethylation, but its administration reduces the length of regenerating axons in mice [64]. Valproic acid has been reported to promote skin wound healing [65] and neuronal repair [66]. In our experiments in the model of ear pinna, the effects of non-nucleoside DNA methylation inhibitors hydralazine and RG108 were not encouraging. Valproic acid, a histone deacetylase inhibitor, at a 500 mg/kg dose induced a modest but statistically significant increase in ear hole closure.

### 2.6. Impact of Mouse Age on Ear Pinna Healing

**Figure 12:**
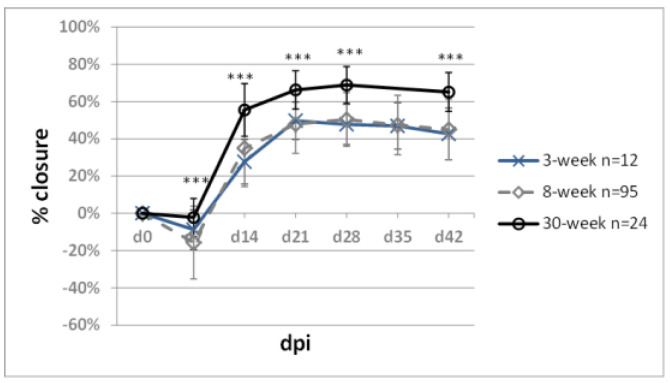
Mouse age and ear pinna hole closure. Triple asterisks (***) denote statistical significance below 0.001. n represents the number of wounds (ears); dpi—days post injury.

Ageing is associated with decreasing regenerative abilities. To address the question of age in mouse ear pinna healing, we compared ear hole closure in females of BALB/c mice at the age of 3, 8, and 30 weeks at the beginning of the experiment, which corresponds to weaned premature, young adult, and middle-aged mice (Figure 12). Significantly increased ear hole closure was observed at all examined time-points in the 30-week-old compared to 8-week-old mice (65.1 ± 10.5% vs. 44.9 ± 16.3). We determined no statistically significant difference in healing between the 3-week-old and 8-week-old mice.

A higher degree of ear pinna hole closure in middle-aged mice was reported by Reines et al. [67], who demonstrated approximately 80% and 50% of ear pinna hole closure 4 weeks post-injury for 8- and 2-month-old BALB/c females, respectively. Our data confirm the finding, but we recorded only 70% closure on day 28 for 8-month-old BALB/c females. Ear pinna hole closure in 3-week-old, weaned premature mice revealed no difference compared to young 8-week-old adults. The age-related changes in ear pinna wound closure indicate that the ear pinna model requires animals of similar age. Also, we found no impact of sexual maturation on ear pinna hole closure. Improved ear pinna healing in 30-week-old compared to 8-week-old mice may appear unexpected, as ageing is associated with declining regenerative capacities. However, it should be stressed that 30-week-old laboratory mice are not considered old—this age in mice can be regarded as the equivalent of 30–40-year-old humans [68].

### 2.7. Correlations of Healing between Left and Right Ears

**Figure 13:**
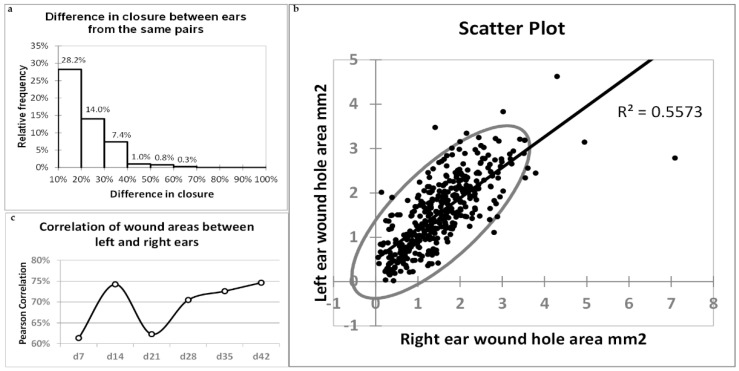
Correlation of wound closure between the left and right ears. (**a**) Histogram demonstrating relative frequencies of percentage differences in ear pinna hole closure between ears from the same mice for 393 animals in the study; (**b**) scatter plot demonstrating wound closure data for 393 ear pairs on day 42 post-injury; (**c**) the Pearson correlation coefficients between the left and right ears determined for 393 ear pairs plotted at progressive time-points.

Infrequently, the percentage of ear hole closure may differ markedly between the ears of the same mouse. Only 9.7% of 393 ear pairs analysed in the present study showed a difference in ear hole closure on day 42 exceeding 30%, and the mean difference for all pairs was 13.7% (Figure 13a). We determined a remarkable (75%) and significant (*p* < 0.0001) correlation in ear pinna hole closure between the left and right ears. The correlation is depicted by a scatter plot (Figure 13b). The correlations determined weekly from day 7 to day 42 post-injury displayed a marked decrease on day 21 to 62.3% compared to day 14 (74.2%), followed by a gradual increase to 74.6% on day 42 (Figure 13c).

The ears of a single mouse are not identical. The differences are particularly accentuated in the patterns of nerve fibres and vessels. Punch wounds are made in the centre of ear pinnae, but their locations relative to the ear base are not perfectly reproducible. This provokes the question of how the differences in ear pinna architecture and wound location may impact ear pinna hole closure. In general, the question is whether the closure results depend more on the microenvironment of individual ears or the organism’s condition as a whole. The healing results were not always similar for two ears from the same mouse. However, a high correlation of closure (74.6%) between the left and right ears indicated that auricle architecture and wound location do not play the leading roles.

### 2.8. Nerve Fibres and Vessels in Regenerating Ear Pinnae

**Figure 14:**
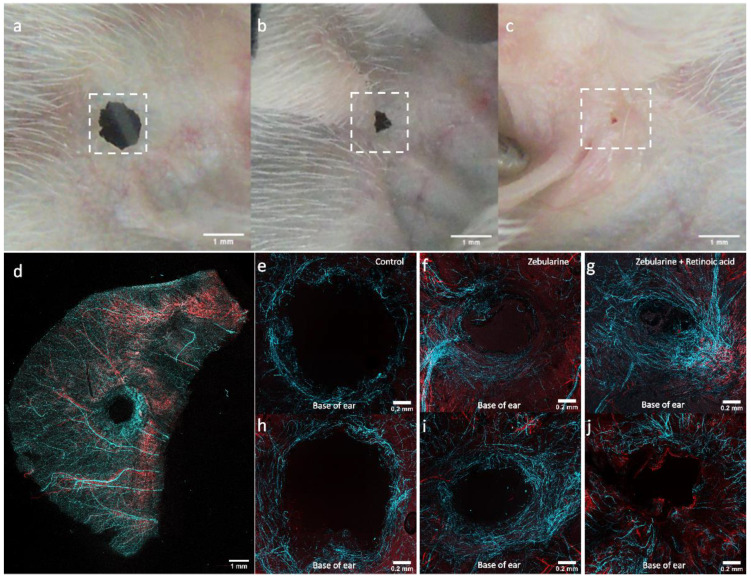
Development of nerve fibres and blood vessels in ear pinnae regenerating following zebularine and retinoic acid treatment on day 42 post-injury. Macroscopic images of ear pinnae used in the examination collected from mice (**a**) receiving saline, (**b**) treated with zebularine alone, and (**c**) treated with zebularine and retinoic acid. The areas selected for microscopic examination are indicated with white squares. (**e**,**h**) Microphotographs (10× objective lens) with immunohistochemical staining for neuron-specific class III ß-tubulin (Tuj1, light blue pseudocolour) and vascular smooth muscle (αSMA, red pseudocolour) of ear pinna wounds for saline-treated controls, (**f**,**i**) mice treated with zebularine alone, (**g**,**j**) and mice treated with zebularine and retinoic acid. The upper panels represent the outer (**e**–**g**) and lower (**h**–**j**) panels the inner aspects of the dissected ear pinnae. (**d**) A whole immunostained ear from the control under a lower magnification (5× objective lens) is shown for comparison.

Observations of wound surface allow the progress of ear pinna hole closure and epithelium formation to be tracked. Microscopic observations reveal the tissue architecture of the regenerating area. The ear pinna model also offers the possibility of examining the growth of nerve fibres and vessels. We performed a series of immunohistochemical stainings to demonstrate a dense network of blood vessels and nerve fibres forming around the ear pinna wound edges and within the restored areas of the ear pinnae. The observations were made in mice treated with zebularine (1000 mg/kg) and retinoic acid (16 mg/kg), zebularine alone, and the controls receiving vehicle on day 42 post-injury (Figure 14). The networks of nerve fibres appeared to expand from the ear’s base (the part of the ear proximal to the head). In the controls, the nerve fibres formed a circular arrangement around the wound, sparsely spreading from the wound margins (Figure 14a,e,h). In the zebularine-treated mice, nerve fibres grew from a major bundle (Figure 14b,f,i). In mice treated with zebularine and retinoic acid, a dense network of nerve fibres penetrated almost the whole regenerated area (Figure 14c,g,j). The newly formed nerve fibre networks in healing ear pinnae were observed on day 42 post-injury in both the control and zebularine- and retinoic acid-treated mice. However, the extent of restored ear pinnae following treatment with zebularine and retinoic acid or zebularine applied alone exceeded that in control (Figure 14b,c vs. Figure 14a), as the extent of newly formed nerve fibres does. Nevertheless, the densities of nerve fibres in the regenerated areas displayed no significant differences (Supplementary File S3).

The growth of nerve fibres in regenerating ear pinnae seems to correspond to the transcriptional induction of genes related to neuronal development, including *Myt1l*, *Neurod1*, *Neurod6*, *Ngf*, *Bdnf*, and *Ntf3*, observed previously in response to zebularine treatment in regenerating ear pinna [34]. Buckley et al. [12] have already shown enhanced growth of nerve fibres in spontaneously healing ear pinnae of the MRL mouse. The present study demonstrated peripheral nerve regeneration in ear pinnae in response to pharmacological stimulation. Although the research on peripheral nerve regeneration often concentrates on sciatic nerve injuries, we demonstrated that an ear pinna model could be helpful in assessing the neuroregenerative potential of pharmaceuticals.

## 3. Materials and Methods

### 3.1. Animals

The experiments on mice were conducted in the Tri-City Academic Laboratory Animal Centre of the Medical University of Gdańsk, where the animals were bred and maintained. The animal study protocols were approved by the Local Ethics Committee for Animal Experimentation in Bydgoszcz (permit No. 5/2015). Animal experimentation was carried out in accordance with the EU directive 2010/63/EU. The experiments were performed on 8-week-old female mice of the BALB/c strain, except for age impact examination, where 5- and 30-week old mice were used.

### 3.2. Ear Pinna Punch Wound Experiment

The mice were anaesthetised before through-and-through holes 2 mm in diameter were made in the mouse ear pinna using a scissor-style ear punch (Fine Science Tools (FST), Foster City, CA, U.S.A., Cat No. 24212-02). Next, the mice were randomly divided into treatment and control groups consisting of six animals. Bioactive agents (Table 2) were administered in intraperitoneal injections, as specified in Table 3, except for vitamin D_3_, which was orally delivered. The control mice received the vehicle alone. The first injection was made immediately after the injury (day 0); if not indicated otherwise, the subsequent injections were made as indicated in Table 2. Ears were photographed weekly starting from the day of the injury, and the wound areas were determined using image analysis with ImageJ software [69]. The ear pinna hole areas are collected in Supplementary File S1.

### 3.3. Fortified Diet Experiment

Vitamins C, D_3_, B_5_, and A were added at 5000, 600, 1.25, and 15 mg, respectively, per 1 kg of unsaturated fatty acid-enriched feed (UFA feed, Altromin C1057 (Lage, Germany). Pellets weighing approximately 3 g were formed using a mechanical press. Mice were given the feed ad libitum for two weeks starting from the day of injury.

### 3.4. Immunohistochemical Analysis

The mice were punched as described above and treated with zebularine and retinoic acid as described in Table 3. On day 42 post-injury, the animals were euthanised, and ear pinnae were collected. The ears were dissected using forceps, acquiring two sides of the ear—outer and inner (closer to the cheek) sides—and each side was fixed in 4% paraformaldehyde in 0.01 M phosphate-buffered saline (PBS) at 4 °C for 1 h. After three washes for 5 min each in 0.01 M PBS, the cartilage layer was gently removed by scrubbing using a small spatula. Next, samples were incubated at 4 °C for 2 h in a blocking buffer comprising 2% BSA and 0.5% Triton X-100 in 0.01 M PBS. Then, the samples were incubated with diluted (1:300) antibodies in the blocking buffer listed earlier at 4 °C overnight with slight agitation. The primary antibodies were Tuj1 conjugated with Alexa-647 to detect neuron-specific class III ß-tubulin (Biolegend, San Diego, CA, U.S.A., Cat. No. 801201) and αSMA conjugated with Cy3 (Merck, Poznań, Poland, Cat. No. C6198) to detect vascular smooth muscle (Table 4). Finally, samples were washed in 0.01 M PBS with 0.2% Triton X-100 3 times for 15 min. Samples were placed on a microscopic glass slide, covered with a mounting medium VectaShield Vibrance (Vector Laboratories, Burlingame, CA, U.S.A., Cat. No. H-1700-10) and a glass coverslip. Microphotographs of the ear pinnae were captured with the confocal microscope Zeiss LSM800 using ZEN 2.6 Software. A picture of the whole ear pinna was taken using a 5× objective lens from 400 µm in depth (5 optical slices). Pictures of the wound area (1.8 mm × 1.8 mm) were taken using a 10× objective lens from 100 μm in depth (6 optical slices). Lasers of 640 nm and 561 nm were used for excitation of Alexa Fluor-647 and Cy3 fluorescent dyes, respectively. Photomicrographs were exported, and a maximum projection of all z-stack functions was used in ImageJ software to obtain one-plane photomicrographs. The morphometric analysis of nerve fibre densities is described in detail in Supplementary File S3.

### 3.5. Statistical Analysis

Two-sample comparisons were performed with the two-tailed Mann–Whitney U test using the exact computation method. Each ear pinna was treated as a single observation. Ear pinna hole closure data (Supplementary File S1) obtained for the animal groups within this work were compared to the results for saline receiving controls and zebularine-treated mice (1000 mg/kg) from our previous work [34], as indicated in Figure 2, Figure 3, Figure 4, Figure 5, Figure 6, Figure 7, Figure 8, Figure 9, Figure 10, Figure 11 and Figure 12. A value of *p* < 0.05 was taken as significant. The Pearson test was used for correlation analysis. The computations were done using XLSTAT (Addinsoft, Paris, France).

## 4. Conclusions

It has been demonstrated that ear pinna hole closure can be induced pharmacologically [20,21,34,70]. This study presents the experiments with a selection of bioactive compounds tested for pro-regenerative activity using the ear pinna model. We observed significant ear pinna hole closure for 4-ketoretinoic acid, valproic acid, and high-dose methionine. These observations may delineate the directions of further studies. Significantly, the finding that methionine, a dietary essential amino acid, can promote regenerative response is stimulating. Our previous finding that a DNA methyltransferase inhibitor zebularine induced ear pinna regeneration provoked the concept that epigenetic inhibitors may activate endogenous regenerative potential [34]. A similar result was obtained for valproic acid, another epigenetic agent, supporting this concept. Epigenetic drugs may raise concerns about the risk of epi-mutations and thus cancers. Valproic acid has a long record of being an antiepileptic drug and has not been associated with an increased risk of cancers [71]. The observation that 4-ketoretinoic acid, a product of retinoic acid bioconversion, promoted ear pinna healing indicates retinoic acid metabolites as promising pro-regenerative agents. 

Several tested bio-active compounds showed no significant effects on ear pinna hole closure in the tested dose and treatment schedule. We think that these negative results are valuable as preliminary observations.

The pharmaceuticals tested in the ear pinna model can be administered topically but also orally and systemically. Topical delivery seems less reliable here than systemic administration due to the small surface of wound edges. The oral and systemic delivery is vital, as ear pinna healing may manifest regenerative responses in different tissues. It is worth noting that the effect of wound healing does not seem to depend strongly on the tissue architecture of individual ears or the reproducibility of performing ear punches. The high correlation between the left and right ears’ wound closures we determined indicates the predominant impact of the organism as a whole.

The comparison of ear pinna hole closure for 3-, 8-, and 30-week-old mice we present indicates age’s impact on ear pinna hole closure. However, it is worth noting that the results do not explain whether the healing responses to pharmaceuticals in ear pinna may differ depending on age.

Furthermore, we demonstrate that ear pinna punch wounds can be a convenient model to investigate the pro-angiogenic and neuroregenerative potential of tested pharmaceuticals. 

The key question remains on the adequacy of the ear pinna model in mice for the pro-regenerative potential of the tested compounds in human tissues. The observations reported in the animal models with remarkable innate regenerative abilities, *Acomys* [29], the MRL, and the nude mouse [17], suggest that ear pinna closure may manifest enhanced potential for regeneration in other organs. No doubt, experimental evidence would be necessary to determine whether pharmacological agents that induce ear pinna healing promote regeneration in other models. Since ear pinna regeneration involves the growth of not a single tissue type but a complex structure consisting of skin, cartilage, muscles, vessels, and nerve fibres, drugs promoting ear pinna healing are worth testing in different organs and injury models.

## Figures and Tables

**Table 1:** Vitamin A-, B_5_-, C-, and D_3_-fortified diet used in the experiment.

Vitamins	Standard Maintenance Feed C1320 (Altromin) (mg/kg Feed)	Unsaturated Fatty Acids-Enriched Feed (UFA Feed) C1057 (Altromin) (mg/kg Feed)	Vitamins Added (mg/kg Feed)	Vitamin-Fortified UFA Feed (mg/kg Feed)	Fortification vs. Standard Diet %
Vitamin A	4.5 *	4.5 *	15	19.5	433%
Vitamin B_5_	21	50	600	650	3095%
Vitamin C	36	20	5000	5020	13,944%
Vitamin D_3_	0.015 **	0.0125 **	1.25	1.2625	8417%

* To convert vitamin A units into milligrams, it was assumed that 1 IU corresponds to 0.3 µg. ** To convert vitamin D_3_ units into milligrams, it was assumed that 1 IU corresponds to 0.025 µg.

**Table 2:** Bioactive compounds used in animal experiments.

Compound	Source	Cat. No.
All-trans 4-keto retinoic acid	TRC (Toronto Research Chemicals, Toronto, Canada)	K204980
Desloratadine	TCI Europe (Tokyo Chemical Industry, Zwijndrecht, Belgium)	D3787
Famotidine	TCI Europe (Tokyo Chemical Industry, Zwijndrecht, Belgium)	F0530
Folic acid	Sigma-Aldrich (Poznań, Poland)	F7876
GM1485	Key Organics (Camelford, UK)	EG-0058
Hydralazine	TCI Europe (Tokyo Chemical Industry, Zwijndrecht, Belgium)	H0409
L-5-methyltetrahydrofolate	Biosynth Carbosynth (Staad, Switzerland)	FM11406
Methionine	Sigma-Aldrich (Poznań, Poland)	M5308
All-trans-retinoic acid	TCI Europe (Tokyo Chemical Industry, Zwijndrecht, Belgium)	R0064
RG108	Synthesis by P. Mucha, University of Gdańsk (Supplementary File S2)	
Tacrolimus	Selleckchem (Houston, TX, U.S.A.)	S5003
Valproic acid	TCI Europe (Tokyo Chemical Industry, Zwijndrecht, Belgium)	S0894
Vitamin B_5_ (D-pantothenic acid)	Sigma-Aldrich (Poznań, Poland)	21210
Vitamin C (L-ascorbic acid)	Sigma-Aldrich (Poznań, Poland)	A0278
Vitamin D_3_ (cholecalciferol)	TCI Europe (Tokyo Chemical Industry, Zwijndrecht, Belgium)	C0314
Zebularine	TCI Europe (Tokyo Chemical Industry, Zwijndrecht, Belgium)	Z0022

**Table 3:** Administration schedules used in the animal experiments.

Compound	Dose	Vehicles	Volume	Admin. Schedule (Injection Days)
Zebularine	1000 mg/kg b.w.	Saline	0.02 mL per gram b.w.	0–4, 7, 10 *
Zebularine	200 mg/kg b.w.	Saline	0.02 mL per gram b.w.	0–4, 7, 10
Saline control		Saline	0.02 mL per gram b.w.	0–4, 7, 10
RG108	10 mg/kg b.w.	Saline + 1% DMSO	0.2 mL	0–4, 7–10
Control for RG108		Saline + 1% DMSO	0.2 mL	0–4, 7–10
All-trans-retinoic acid	16 mg/kg b.w.	Rapeseed oil + 10% DMSO	0.2 mL	0–4, 7–11 or 0, 2, 4, 7, 9, 11, 14, 16, 18 **
Control for retinoids		Rapeseed oil + 10% DMSO	0.2 mL	0–4, 7, 11
Hydralazine	10 mg/kg b.w.	Saline	0.01 mL per gram b.w.	0–4, 7, 10
Valproic acid	25 mg/kg b.w.	Saline	0.02 mL per gram b.w.	0–4, 7, 10
	500 mg/kg b.w.	Saline	0.02 mL per gram b.w.	0–4, 7, 10
Famotidine	0.5 mg/kg b.w.	Saline	0.02 mL per gram b.w.	0–4, 7, 10
Desloratadine	0.5 mg/kg b.w.	Saline	0.02 mL per gram b.w.	0–4, 7, 10
Tacrolimus	0.25 mg/kg b.w.	Saline	0.4 mL	0–4, 7, 11
GM1485	5 mg/kg b.w.	Saline	0.2 mL	0–4, 7, 11
Famotidine + zebularine	0.5 mg/kg b.w. + 1000 mg/kg b.w.	Saline	0.02 mL per gram b.w.	0–4, 7, 10
Desloratadine + zebularine	0.5 mg/kg b.w. + 1000 mg/kg b.w.	Saline	0.02 mL per gram b.w.	0–4, 7, 10
Zebularine + folic acid + L-5-methyltetra-hydrofolate	1000 mg/kg b.w. + 0.16 mg/kg b.w. + 0.08 mg/kg b.w.	Saline	0.02 mL per gram b.w.	0–4, 7, 10
Folic acid + L-5-methyltetra-hydrofolate	0.16 mg/kg b.w. 0.08 mg/kg b.w.	Saline	0.02 mL per gram b.w.	0–4, 7, 10
Folic acid	0.08 mg/kg b.w.	Saline	0.2 mL	0–4, 7–11
L-5-methyltetra-hydrofolate	0.08 mg/kg b.w.	Saline	0.2 mL	0–4, 7–11
Vitamin C (ascorbic acid)	1%	Saline	0.2 mL	0–4, 7–11
Methionine	125 mg/kg b.w.	Saline	0.02 mL per gram b.w.	0–4, 7, 10
Vitamin D_3_	50 IU	Rapeseed oil	0.1 mL	0–4, 7–11
Zebularine + Retinoic acid ***	1000 mg/kg b.w. 16 mg/kg b.w	Saline Rapeseed oil, 0.3% DMSO	0.02 mL per gram b.w. 0.2 mL	0–4, 7, 10 0, 2, 4, 7,9, 11
Control for zebularine and retinoic acid ***		Saline Rapeseed oil, 0.3% DMSO	0.02 mL per gram b.w. 0.2 mL	0–4, 7, 10 0, 2, 4, 7,9, 11

* For delayed zebularine administration, the start of experiments days was shifted to day 3 or day 14, as indicated in Figure 2. ** Extended retinoic acid administration for 3 weeks, as indicated in Figure 7b. *** Zebularine and retinoic acid administration for immunohistochemistry.

**Table 4:** The primary antibodies used for immunohistochemistry.

Antibody	Marker	Conjugated	Host	Clonality and Isotype	Supplier, Cat. Number
Tuj1	III β-tubulin	Alexa Fluor 647	Mouse	Monoclonal, IgG2a	Biolegend, 801201
αSMA	Alfa smooth muscle actin	Cy3	Mouse	Monoclonal, IgG2a	Merck, C6198

## Data Availability

Data is contained within the article and Supplementary Materials.

## Supplementary Materials

The following supporting information can be downloaded at https://www.mdpi.com/article/10.3390/ph15050610/s1: Supplementary File S1—ear pinna hole closure data, Supplementary File S2—description of RG108 synthesis, Supplementary File S3—morphometric analyses of nerve fibres.
