# Supplementary material for: Regenerative Drug Discovery Using Ear Pinna Punch Wound Model in Mice
Source: Pharmaceuticals (Basel). 2022 May 16;15(5):610. doi: 10.3390/ph15050610 (PMC9145447; doi:10.3390/ph15050610)
Supplement: Supplementary file 1 [file pharmaceuticals-15-00610-s001.zip › Supplementary File S2. Description of RG108 synthesis.pdf]

Sosnowski *et al.* 2022, Regenerative Drug Discovery Using Ear Pinna Punch Wound Model in Mice.

Supplementary File S2.

Synthesis of *N*-phthaloyl-L-tryptophan (RG108)

A mixture of L-tryptophan (0.01 mmol) and phthalic anhydride (0.01 mmol) was suspended in *N,N*-dimethylformamide (DMF, 20 ml). Following trimethylamine (TEA, 0.01 mmol) was added. The mixture was heated under reflux at 100 ^{o}C for 5 hours. The crude reaction mixture was diluted with ethyl acetate (20 ml) and washed with brine (3x50 ml). The organic layer was dried using magnesium sulfate, filtrated, and the solvent was removed in vacuo. Crude RG108 was purified by flash chromatography using a silica column and a dichloromethane (DCM): methanol (MeOH): acetic acid (AcOH) (95:4:1 v/v/v) mixture as eluent. Yield 68%.

Analysis data

RGD108 homogeneity and composition were confirmed by analytical RP HPLC method (Agilent1200 chromatograph, Kinetex XB-C18 column, 150x4,6mm, 5 µm particle; solvents A-0.08% TFA, B-ACN (0.08% TFA), linear gradient 40-80% B in 30 min, detection at 254 nm, flow 1 ml/min) and mass spectrometry (Sciex QTOF 5600+ spectrometer, normal polarity (M+H calc. 335.33, obs. 335.13)). The results of RP HPLC and MS are shown below.

RP HPLC

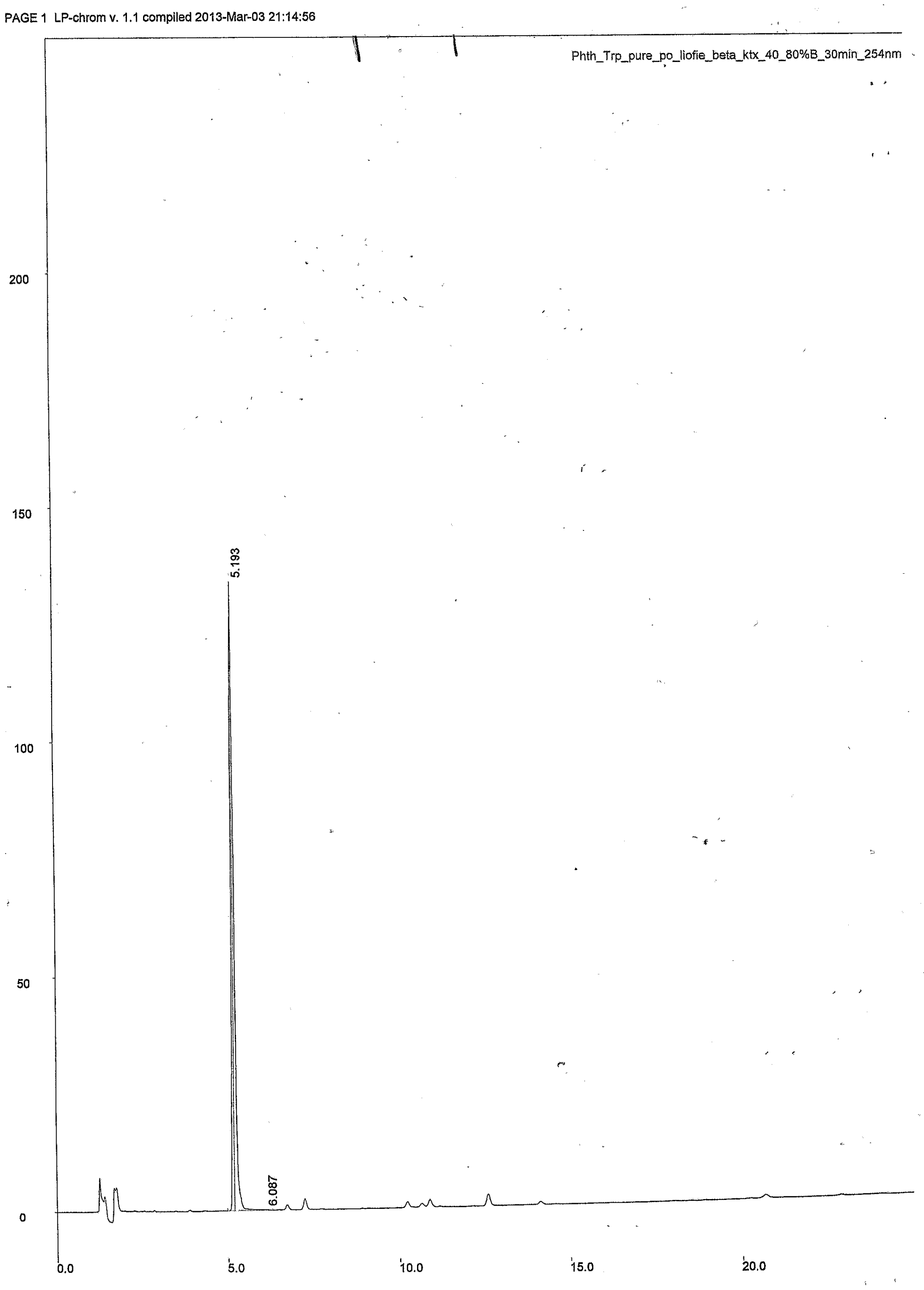
PAGE 1 LP-chrom v. 1.1 compiled 2013-Mar-03 21:14:56
Phth_Trp_pure_po_liofie_beta_ktx_40_80%B_30min_254nm
200
150
100
50
0
5.193
6.087
0.0
5.0
10.0
15.0
20.0

MS

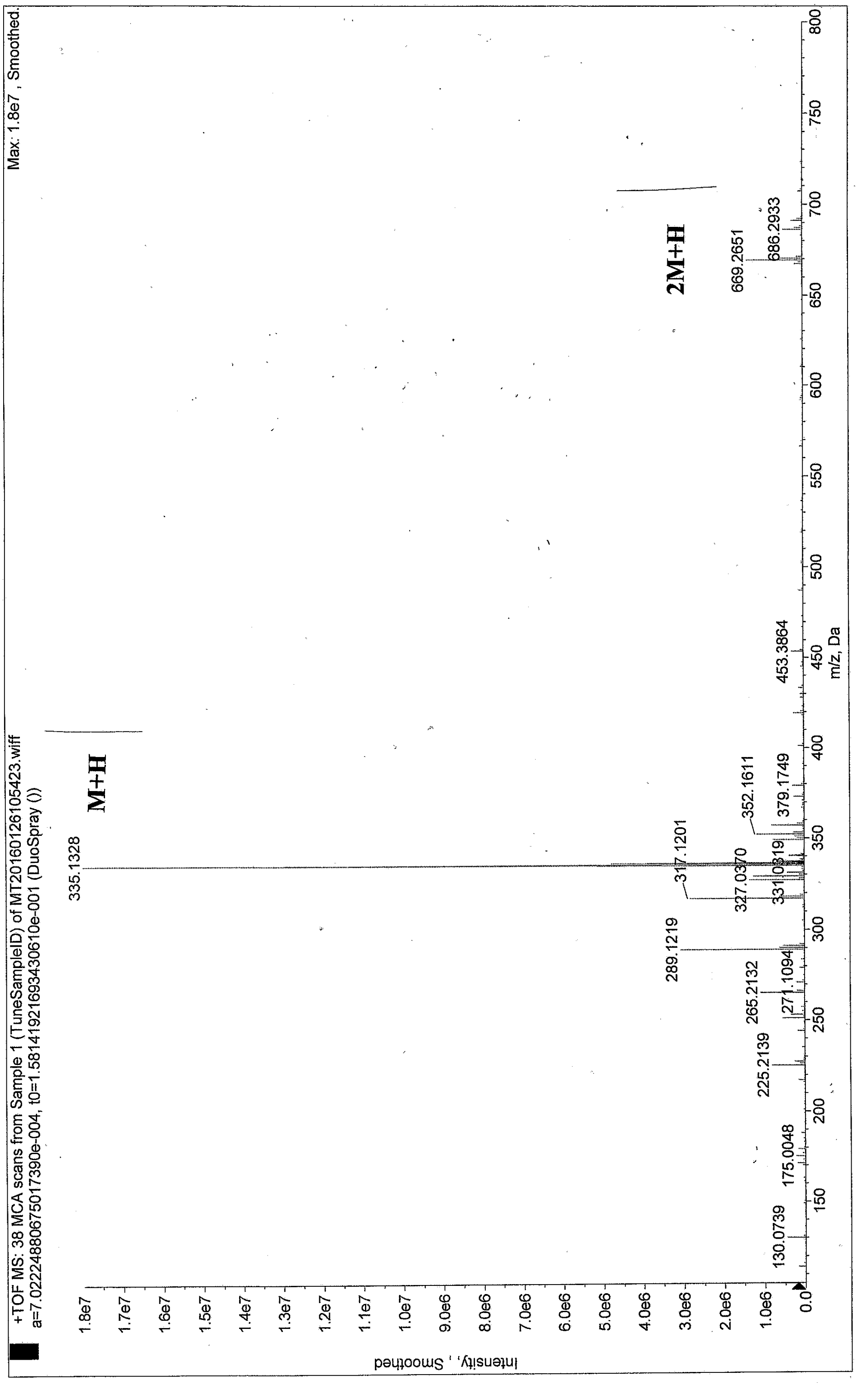
+TOF MS: 38 MCA scans from Sample 1 (TuneSampleID) of MT20160126105423.wiff
a=7.02224880675017390e-004, t0=1.58141921693430610e-001 (DuoSpray ())
Max. 1.8e7 , Smoothed.
Intensity, , Smoothed
m/z, Da
M+H
2M+H
335.1328
317.1201
289.1219
327.0370
331.0319
352.1611
379.1749
453.3864
265.2132
271.1094
225.2139
175.0048
130.0739
669.2651
686.2933
