# Supplementary material for: Regenerative Drug Discovery Using Ear Pinna Punch Wound Model in Mice
Source: Pharmaceuticals (Basel). 2022 May 16;15(5):610. doi: 10.3390/ph15050610 (PMC9145447; doi:10.3390/ph15050610)
Supplement: Supplementary file 1 [file pharmaceuticals-15-00610-s001.zip › Supplementary File S3. Morphometric analysis of nerve fibres.df.pdf]

Sosnowski *et al.* 2022, Regenerative Drug Discovery Using Ear Pinna Punch Wound Model in Mice.

Supplementary File S3. Description and results of morphometric analysis of the regenerating nerves in ear pinnae.

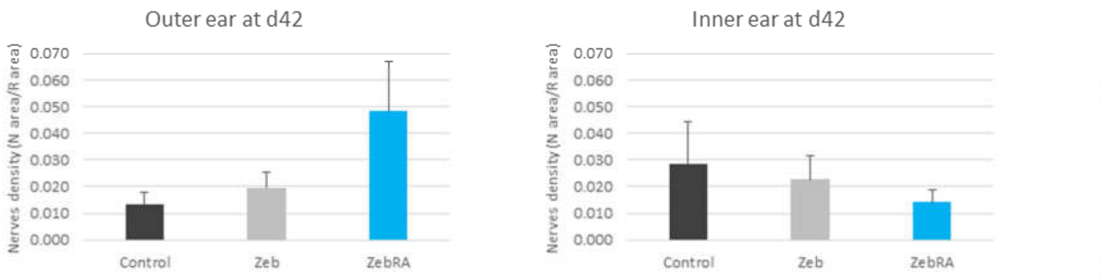

Suppl. File S3. The effect of zebularine 1000 mg/kg (Zeb) and zebularine with retinoic acid (ZebRA; Zeb - 1000 mg/kg, RA 16 mg/kg) compared to saline (Control) on nerves density in the regenerated areas of the outer and inner aspects of the ear pinnae. N area – nerves area; R area – regenerated area. Error bars indicate SD.

Morphometric analysis of nerves density

Photomicrographs of ear pinnae's outer and inner aspects were collected as described in the "*3.4. Immunohistochemical analysis"* paragraph of Materials and Methods. Three consecutive optical slices with the most abundant nerve signal for each photograph were chosen and exported to ImageJ software for analysis. The pictures were calibrated, wound edges were manually drawn, and residual wound area (W area) was calculated. Next, the regenerated area (R area) was determined by subtracting the W area from the original injury area ($3.14\ mm^2 = 3\ 140\ 000\ um^2$). The pictures were subjected to background subtraction to minimise autofluorescence input. The nerves area (N area) was calculated by manual thresholding. Background correction and thresholding were precisely the same for all pictures analysed. Finally, the density was calculated by dividing N area by R area for each optical slice, and the results represent the mean density from three pictures for each condition analysed.
